# Detection of Hepatitis B virus subgenotype A1 in a Quilombo community from Maranhão, Brazil

**DOI:** 10.1186/1743-422X-8-415

**Published:** 2011-08-25

**Authors:** Mónica V Alvarado-Mora, Livia Botelho, Michele S Gomes-Gouvêa, Vanda F de Souza, Maria C Nascimento, Claudio S Pannuti, Flair J Carrilho, João RR Pinho

**Affiliations:** 1 Laboratory of Tropical Gastroenterology and Hepatology, São Paulo Institute of Tropical Medicine and Department of Gastroenterology, School of Medicine, University of São Paulo, São Paulo, Brazil; 2 Laboratory of Virology, São Paulo Institute of Tropical Medicine, Department of Infectious and Parasitic Diseases, School of Medicine, University of São Paulo, Brazil

**Keywords:** Hepatitis B virus, Genotype A1, Quilombo community, Maranhão state, Brazil, Bayesian Analysis

## Abstract

**Background:**

The Brazilian population is mainly descendant from European colonizers, Africans and Native Americans. Some Afro-descendants lived in small isolated communities since the slavery period. The epidemiological status of HBV infection in Quilombos communities from northeast of Brazil remains unknown. The aim of this study was to characterize the HBV genotypes circulating inside a Quilombo isolated community from Maranhão State, Brazil.

**Methods:**

Seventy-two samples from Frechal Quilombo community at Maranhão were collected. All serum samples were screened by enzyme-linked immunosorbent assays for the presence of hepatitis B surface antigen (HBsAg). HBsAg positive samples were submitted to DNA extraction and a fragment of 1306 bp partially comprising HBsAg and polymerase coding regions (S/POL) was amplified by nested PCR and its nucleotide sequence was determined. Viral isolates were genotyped by phylogenetic analysis using reference sequences from each genotype obtained from GenBank (n = 320). Sequences were aligned using Muscle software and edited in the SE-AL software. Bayesian phylogenetic analyses were conducted using Markov Chain Monte Carlo (MCMC) method to obtain the MCC tree using BEAST v.1.5.3.

**Results:**

Of the 72 individuals, 9 (12.5%) were HBsAg-positive and 4 of them were successfully sequenced for the 1306 bp fragment. All these samples were genotype A1 and grouped together with other sequences reported from Brazil.

**Conclusions:**

The present study represents the first report on the HBV genotypes characterization of this community in the Maranhão state in Brazil where a high HBsAg frequency was found. In this study, we reported a high frequency of HBV infection and the exclusive presence of subgenotype A1 in an Afro-descendent community in the Maranhão State, Brazil.

## Introduction

It is estimated that two billion people have been infected with Hepatitis B virus (HBV) and that more than 350 million are chronic carriers of this virus [1]. HBV strains have distinct geographical distribution and are classified into nine genotypes, A to I, on basis of genome diversity [2]. Genotype A is found mainly in North America and Africa and has been found in six genetically distinct subgenotypes (A1 to A6) [3]. Genotypes B and C are prevalent in Southeast Asia and the Far East. Genotype D has a worldwide distribution and is found predominantly in the Mediterranean region and Central Asia. Genotypes E and F are prevalent in West Africa and in the Native American population, respectively [4]. In addition, genotype G has been reported in the USA, France [5], Colombia [6] and Brasil [7] and genotype H has been found in North and Central America [8]. Recently, by using phylogenetic analyses, a new genotype was characterized in Vietnam and Laos and designated as genotype I [2,9,10].

In Brazil, a wide variation of HBV infection prevalence was reported, particularly dependent upon the geographical region of this country [11]. Genotype A is the most prevalent and genotypes B, C, D and F are also circulating in the population [12-15]. The presence of these genotypes reflects the mixture of cultures in the Brazil: Native American, European and African ancestral roots, showing this country as an important model for studies of population genetics [16].

During the slavery period in Brazil (from XVI to XIX centuries), some African people managed to escape to refuge areas, living with others in well hidden places in the woods. These places were known as Quilombos and they were regions of large concentration of runaway-slaves, far from urban centers and located in areas with difficult access. Their inhabitants generally stayed in culturally isolated communities without significant additional admixture since their establishment. Quilombos were located in different Brazilian states around the country: Pará (PA), Maranhão (MA), Alagoas (AL), Pernambuco (PE), Bahia (BA), Goiás (GO), Mato Grosso do Sul (MS), Minas Gerais (MG), Rio de Janeiro (RJ) and São Paulo (SP) [17].

**Figure 1:**
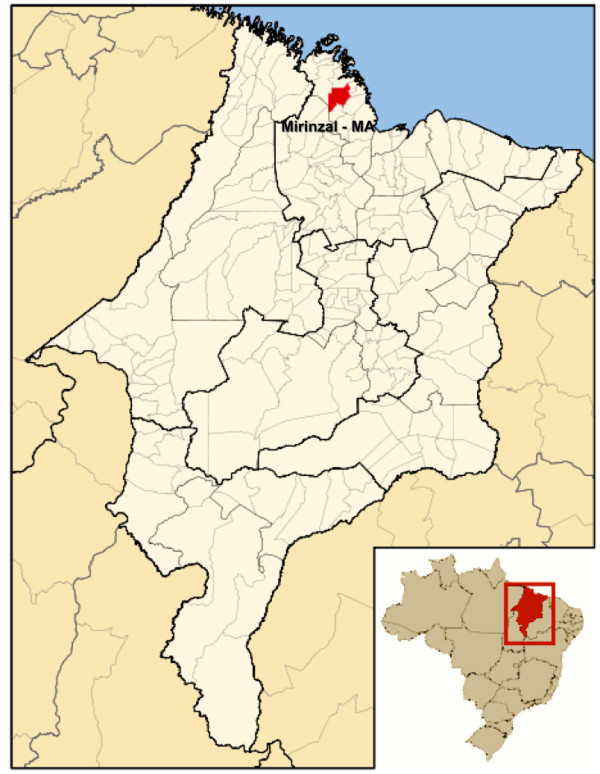
**Geographical localization of the Afro-Brazilian community in Maranhão State - Brazil (modified of http://pt.wikipedia.org/wiki/Ficheiro:Maranhao_Municip_Mirinzal.svg)**.

The quilombo Frechal is located in the municipality of Mirinzal, at the region of lower western Maranhão State, Brazil (Figure 1). The Frechal community was founded in the late XVIII century and was devoted to sugar production till the XIX century. The Frechal community is one of the oldest and most important Quilombos located in Maranhão State (http://www.cpisp.org.br/comunidades/html/brasil/ma/ma_comunidades_frechal.html, accessed at 11/01/2011).

The aim of this study was to analyze the presence of current HBV infection by HBsAg and HBV DNA detection in the current inhabitants of Frechal community and to determine which HBV genotypes are found among these cases.

## Materials and methods

### Study Population

Seventy-two samples from inhabitants from Frechal, Maranhão were collected from 2000 to 2001 (Figure 1). Ethical Committee of the University of Sao Paulo, Medical School, Sao Paulo, Brazil, approved this protocol. All patients have signed an informed consent form before the samples were collected for research. The samples were screened for HBsAg using commercially available kits (DiaSorin Ltda, Saluggia, Italy). HBsAg positive samples were submitted to PCR amplification to detect HBV DNA.

### HBV DNA extraction

Viral nucleic acids (HBV DNA and HDV RNA) extraction was carried out from 100 μl of sera for each sample using the acid guanidinium thiocyanate/phenol/chloroform method [18]. To avoid false-positive results, strict procedures for nucleic acid amplification diagnostic techniques were followed [19].

### HBV PCR Amplification

Samples were first amplified with the primers described by Sitnik et al. [13] in order to obtain a 416 base pairs (bp) fragment partially covering the HBsAg coding region (S) to confirm the presence of HBV-DNA in the sample.

To characterize HBV genotypes, a fragment of 1306 bp partially comprising HBsAg and the polymerase coding regions (S/POL) of HBV genome was amplified by nested PCR using the primers PS3132F (3132 nt - 3151 nt)/2920R (1417 nt - 1398 nt) and PS3201F (3201 nt - 3221 nt)/P1285R (1285 nt - 1266 nt) [6].

### HBV Nucleotide Sequencing

Amplified DNA was purified using ChargeSwitch^® ^PCR Clean-Up Kit (Invitrogen, São Paulo, Brazil). Sequencing was performed in an ABI Prism^® ^377 Automatic Sequencer (Applied Biosystems, Foster City, CA, USA), based on the protocol described by Sanger et al. [20], using dideoxy nucleotide triphosphates (ddNTPs) containing fluorescent markers (*Big Dye^® ^Terminator v3.1 Cycle Sequencing Ready Reaction kit *- Applied Biosystems, Foster City, CA, USA). The quality of each electropherogram was evaluated using the Phred-Phrap software [21] and consensus sequences were obtained by using an alignment constructed with CAP3 software available at the web page *Electropherogram quality analysis *(http://asparagin.cenargen.embrapa.br/phph/).

### HBV Genotyping Analysis

Sequences were assigned to HBV genotypes after phylogenetic analysis using reference sequences from each HBV genotype obtained from the GenBank (n = 320). These sequences comprised partial HBsAg and polymerase coding regions (S/POL). They were aligned using Muscle Software [22] and edited with the SE-AL software (available at http://tree.bio.ed.ac.uk/software/seal/). To perform the phylogenetic analysis, the missing nucleotides were coded as "missing characters" in nexus block. Bayesian phylogenetic analyses were done applying Markov Chain Monte Carlo simulation using BEAST v.1.5.3 [23], and 10 million generations were sufficient to obtain the convergence of parameters. The relaxed uncorrelated log_normal _was the best molecular clock for the dataset. The maximum clade credibility (MCC) tree was obtained from summarizing the 10,000 substitution trees and then it was removed 10% of burn-in using Tree Annotator v.1.5.3 [23].

## Results

Of the 72 samples, 9 (12.5%) were HBsAg-positive. Since we did not have epidemiological data about this population, it was not possible to compare HBsAg results with other demographic information. Of these nine samples, six were positive by nested PCR for the S fragment (416 bp) and among them, 4 also amplified the S/POL region (1306 bp).

**Figure 2:**
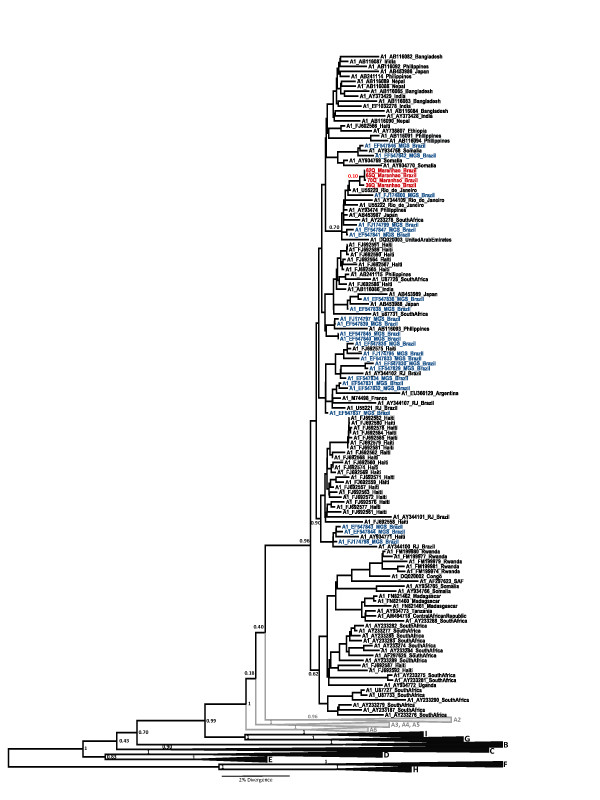
**The maximum clade credibility (MCC) tree was estimated by Bayesian analysis of 320 S/POL sequences with 1306 bp of Hepatitis B virus strains**. The posterior probabilities of the key nodes (internal nodes) are depicted above the respective nodes. Samples HBV/A1 obtained from Frechal (n = 4, red taxa) were analyzed together with other worldwide strains by Maximum Likelihood (ML) method (n = 134). The clusters containing the strains of others Afro-Brazilian communities previously reported are highlighted (Blue taxes). Genotype B (n = 19), genotype C (n = 20), genotype D (n = 24), genotype E (n = 7), genotype F (n = 25), genotype G (n = 4), genotype H (n = 6) and genotype I (n = 9) branches were collapsed. Also, the subgenotypes A2 (n = 52), A3, A4, and A5 (n = 10), A6 (n = 3) and A7 (n = 7) branches were collapsed.

To perform the phylogenetic analysis, the longest fragment available from each sample was sequenced and classified as subgenotype A1 (subtype *adw2*). Although the four samples from Frechal were grouped in the same cluster in the tree, it cannot be assumed that there was a founder effect in this community because this group is supported only with a low posterior probability (0.10) (Figure 2). Sequences were deposited in GenBank at accession numbers: HM772994 - HM772997.

## Discussion

This is the first study that characterized HBV genotypes present in an isolated community from Maranhão state, Brazil. Since the genotype A1 has been reported as a common genotype in African and Brazilian populations [12,13,24-28], the four samples were compared with others previously reported sequences from Quilombos in Brazil [28] and Venezuela [29] and with sequences from Rio de Janeiro, as in this place there was a constant slave traffic from Africa that was most intense between 1795 to 1811 [30]. The subgenotype A1 sequences from Quilombos and Rio de Janeiro did not assemble in a single group in the tree. These results suggest the presence of the different A1 strains within the Quilombos populations. These variants may have come from Africa before these groups were created and actually the HBV/A1 strains continued to evolve in the Afro-descendent population after they came to Brazil.

A lot of Afro-descendants live in Bahia in Northeast Brazil. The geographic distributions of HBV genotypes have showed that genotype A (subtype *adw2*) is the most frequent in according to the ethnic background of the population [31]. It was reported a high prevalence of genotype A in Bahia but short-length sequences available prevented performing phylogenetic analysis together with the other ones analyzed in this study. Moreover, it is possible that different A1 variants have different African origin, as during the slave trade time, slaves from different countries from Africa were mixed on the boats and then were sold and distributed in different Brazilian regions [32].

**Figure 3:**
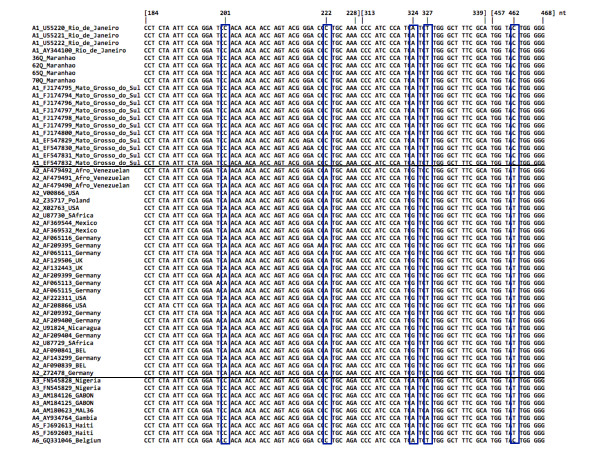
**Multiple alignment of partial HBV/S gene (184 to 468 nt) comprising HBV/A1 sequences from Brazil, which are compared with other sequences of subgenotypes HBV/A2 to HBV/A6 previously reported**. The five specific substitutions for subgenotype A1 are shown in the figure (blue squares)

We found that the three sequences from Afro-Venezuelan population [29] were classified as subgenotype A2 (Figure 3), which suggest different origin of HBV strains circulating in this population compared with African descendants in Brazil. Moreover, a specific polymorphism was found in HBV S region that distinguish subgenotypes A1 and A2 and it was agreed with HBV genotype A classification previously reported [33]. The synonymous substitutions at nucleotides in the third-positions: 201 nt (TC**C **→ TC**A**) Serine [S]; 222 nt (CC**C **→ CC**A**) Proline [P]; 324 nt (TC**A **→ TC**G**) Serine [S]; 327 nt (TC**T **→ TC**C) **Serine [S] and 462 nt (TA**C **→ TA**T) **Tryptofan [W] were identified and they confirm the results of subgenotype classification (Figure 3).

Finally, we found a high frequency of HBsAg in the Frechal population (12.5%). Kalunga population, the largest Afro-Brazilian isolated community located in Goiás state, showed lower frequency for HBsAg (1.8% - 16/878). HBV subgenotype (A1) was also found in all the positive samples from Goiás [34].

Another study, with 1058 individuals living in12 different isolated Afro-descendant communities, was carried out in Mato Grosso do Sul state and showed that among 1058 individuals, 23 (2.2%) of them were HBsAg positive. The highest prevalence was detected in Furnas dos Dionisios community (42.4% to anti-HBc and 7.4% to HBsAg) and the overall prevalence for HBV infection was 19.8% [35]. Subgenotype A1 was the most frequent in this state, followed by subgenotype A2 and genotype D [28].

HBsAg frequency in Afro-Venezuelan communities (3.6%) and in rural populations from Venezuela were higher than those found in Brazilian studies involving Quilombo inhabitants (2.2%) [29,35].

In Frechal, seventy-two samples were collected and this sample size probably did not represent the population but our data suggest a high frequency of HBV in this community. However, further studies are needed to better evaluate the epidemiology of hepatitis B in this region.

In this study, we reported a high frequency of HBV infection and the exclusive presence of subgenotype A1 in an Afro-descendent community in the Maranhão State, Brazil. In conclusion, HBV subgenotype A1 was found in all African descendant population from South America studied so far except in Venezuela, where subgenotype A2 was found. Also, this study shows the need to report more HBV sequences from Afro-descendant communities to complement the actual data and the establishment of an accurate substitution rate for this virus to understand the evolutionary origins. The study of HBV sequences from other Afro-descendant communities from American countries, as well as from other different African countries, will allow a better understanding on HBV spreading between these two continents.

## Competing interests

The authors declare that they have no competing interests.

## Authors' contributions

MVAM participated in the design of the study, conducted the phylogenetic and evolutionary analysis, drafted the manuscript and in its design and coordination. LB participated in the PCR amplification and sequencing process. MSGG participated in the PCR amplification. VFS, MCN, CSP and FJC participated in the design of the study. JRRP participated in the design of the study and drafted the manuscript. All authors read and approved the final manuscript.
